# The Moral Self-Image Scale: Measuring and Understanding the Malleability of the Moral Self

**DOI:** 10.3389/fpsyg.2015.01878

**Published:** 2015-12-15

**Authors:** Jennifer Jordan, Marijke C. Leliveld, Ann E. Tenbrunsel

**Affiliations:** ^1^Department of Human Resource Management & Organizational Behaviour, University of GroningenGroningen, Netherlands; ^2^Department of Marketing, University of GroningenGroningen, Netherlands; ^3^Department of Management, University of Notre DameNotre Dame, IN, USA

**Keywords:** ethics, morality, self-image, self-concept, the self

## Abstract

Recent ethical decision-making models suggest that individuals' own view of their morality is malleable rather than static, responding to their (im)moral actions and reflections about the world around them. Yet no construct currently exists to represent the malleable state of a person's moral self-image (MSI). In this investigation, we define this construct, as well as develop a scale to measure it. Across five studies, we show that feedback about the moral self alters an individual's MSI as measured by our scale. We also find that the MSI is related to, but distinct from, related constructs, including moral identity, self-esteem, and moral disengagement. In Study 1, we administered the MSI scale and several other relevant scales to demonstrate convergent and discriminant validity. In Study 2, we examine the relationship between the MSI and one's ought versus ideal self. In Studies 3 and 4, we find that one's MSI is affected in the predicted directions by manipulated feedback about the moral self, including feedback related to social comparisons of moral behavior (Study 3) and feedback relative to one's own moral ideal (Study 4). Lastly, Study 5 provides evidence that the recall of one's moral or immoral behavior alters people's MSI in the predicted directions. Taken together, these studies suggest that the MSI is malleable and responds to individuals' moral and immoral actions in the outside world. As such, the MSI is an important variable to consider in the study of moral and immoral behavior.

## Introduction

Evidence of unethical behavior is widespread in society. From violations of psychological contracts (e.g., Kotter, 1973) to lying and deception (e.g., Lewicki, 1983), various forms of unethical behavior permeate modern life, creating both economic, and reputational costs. For many years, most empirical research on morality was dominated by the notion that there were stable, individual differences in moral behavior (e.g., Kohlberg, 1971; Colby et al., 1983; Kohlberg et al., 1983). However, contrary to the popular view that unethical behavior is just a matter of “a few bad apples,” a rich body of recent literature demonstrates that even people who care about being moral (that is, those who have a high moral identity; Aquino and Reed, 2002) often engage in unethical behavior (for a recent review, see Bazerman and Gino, 2012). This research also argues that individuals' own perceptions of their morality is dynamic and malleable, and can influence subsequent behavior (Goldstone and Chin, 1993; Monin and Jordan, 2009; Shalvi et al., 2015): at any moment in time, social and situational factors may swing one's moral self-view. In the current investigation, we propose the construct of the *moral self-image* (MSI), defined as a person's dynamic and malleable moral self-concept, to provide insight into this malleability of moral self-perceptions. We also propose a scale to measure the MSI. Across five studies, we demonstrate that this scale responds to feedback from the social world and people's reflections of their own moral behavior. By proposing the construct of the MSI, we hope to clarify how social and intrapersonal events, such as ethical and unethical behavior, shape people's views of their moral selves and how the state of their moral selves can affect their subsequent behaviors.

## The dynamic and malleable nature of morality

People engage in unethical actions on a daily basis, much more often than they care to admit (DePaulo et al., 1996; Schweitzer et al., 2004; Mazar et al., 2008; Gino et al., 2009; Shalvi et al., 2011). At the same time, they strive to maintain a positive self-concept both privately and publicly (Allport, 1955; Jones, 1973; Rosenberg, 1979; Adler, 2006). In fact, people wish to view themselves as moral beings (Steele, 1988; Dunning, 2007; Monin and Jordan, 2009) and take steps to maintain this belief when they behave immorally (Mazar et al., 2008; Monin and Jordan, 2009; Barkan et al., 2012; Shalvi et al., 2015)—even when these steps involve sacrificing gains or investing valuable resources (Murnighan et al., 1993; Dunning, 2007). According to recent research, when people act morally, their self-perception of their own morality is strengthened, allowing them to relax their subsequent moral strivings and engage in immoral actions. In contrast, after individuals act immorally, they seek to strengthen this self-concept by engaging in moral actions (Sachdeva et al., 2009; Jordan et al., 2011). Thus, the extent to which one's perceived morality “measures up” appears to be an important influence on actual (im)moral behavior.

This apparent discrepancy between people's perceived actual and ideal MSI leads to a dynamic and malleable perception of one's moral self: at any given moment, individuals may answer the question “How moral am I?” differently (Monin and Jordan, 2009; Moore and Gino, 2013). We label the answer to this question as a description of a person's MSI.

## Research calling for the MSI^1^

Within the rich body of research on the “self” (both the general and moral self), significant research proposes that the dynamics of the moral self explain immoral and moral behavior, yet no validated tool has been provided to measure this process (Zhong and Liljenquist, 2006; Sachdeva et al., 2009; Barkan et al., 2012; Mulder and Aquino, 2013).

For example, in their research on moral cleansing, Zhong and Liljenquist (2006) discuss the need for people to cleanse the moral self following an immoral act due to the need to self-complete via symbolic actions (Wicklund and Gollwitzer, 1981). The authors discuss that the need to do a good deed following a bad one is motivated by a desire for “restoration or completion of the moral self” (p. 1452); however, they do not measure the moral self nor provide empirical evidence that engaging in this type of deed actually affects people's moral selves.

In a similar vein, Mulder and Aquino (2013) demonstrate that people—particularly those with a high centrality of moral traits (i.e., a high internalized moral identity)—engage in behaviors that help to, “maintain their self-image as a moral person in the aftermath of a dishonest act” (p. 219). Mulder and Aquino find that following cheating, people who hold moral traits to high self-importance will engage in compensatory moral behavior. Although, they do not measure actual changes in one's self-image, they propose that this pattern is a consequence of a desire to “uphold a moral self-image” (p. 219) and reduce the discomfort of violating one's MSI.

Sachdeva et al. (2009) suggest that a person's need to boost the MSI (or what they term the “moral self-worth” and “moral self-concept”) is responsible for compensatory patterns of moral behavior: “That is, when moral self-worth is threatened, moral cleansing restores the moral self-concept, and when moral self-worth is too high, moral licensing allows the agent to restrict moral behavior and return to a more comfortable level” (p. 524). Across a series of three studies, they find that when people write stories about themselves that affirm or threaten their MSI, they then act in opposing directions on subsequent tasks: a flattering story is followed by less moral behavior and an unflattering story is followed with more moral behavior. They also find that these effects do not occur if the story is written about someone else, suggesting that it is moral *self* -image that is at play, though this possibility is not empirically explored.

In a nuanced examination of the influences on dishonest behavior, Mazar et al. (2008) propose that people will be dishonest for self-gain—but only to the extent that dishonesty does not threaten their MSI. Mazar and colleagues use several paradigms in which people have the opportunity to cheat. Across five studies, they find that people *do* cheat—not always in a way that maximizes self-gain, but always in a way that, as they argue, protects their cherished MSI. For example, the researchers find that when dishonesty is framed in a way that makes a person mindful of her moral self-standards, she refrains from cheating in an effort to preserve her MSI. Mazar and colleagues argue that prior theories of dishonesty have failed to account for the value people place on maintaining their MSI, instead favoring a viewpoint that emphasizes a cost-benefit analysis on the part of the cheater. The researchers attempted to identify actual changes to participants' MSI by asking them about how moral they view themselves to be, but these questions yielded no effects.

We see a similar emphasis on MSI as an explanatory process in recent work that utilizes a cognitive dissonance framing to explain the effects of behavior on people's MSI (Shalvi et al., 2015). Barkan et al. (2012) demonstrated that having people contemplate their immoral misdeeds subsequently lowered their state self-esteem (Heatherton and Polivy, 1991; which then explained their greater willingness to punish and negatively judge other wrong-doers). Across six studies, they found that having people think about an unethical behavior that produced guilt, shame, or regret led participants' to report lower general self-images (compared to recalling neutral or favorable situations about the self). However, the authors also found that recalling a domain-general personal failure or an amoral behavior that elicited cognitive dissonance produced the same lowered state self-esteem. We suspect that had the authors specifically measured participants' MSI, changes would have only occurred in response to the immoral recall (see Studies 4 and 5 in the current manuscript for support for this supposition).

Lastly, Monin and Jordan (2009) discussed the emergence of a construct that captured the dynamics of the moral self. In their theoretical piece they discuss “a view of the self that is more reflective and more labile—one's moment to moment question of ‘How moral am I?”’ (p. 347), a question that they say people constantly strive to answer favorably. They explicitly call for a tool to measure the mechanism between an individual and his or her behavior, saying that understanding the dynamics of the moral self will “broad[en] the scope of phenomena that can be studied” (p. 348).

The literature reviewed above proposes the dynamics of the MSI as a mechanism for the dynamics of moral and immoral behavior. Yet because no tool is provided to empirically measure the state of the moral self and its dynamics, these assertions lack empirical evidence. One exception is a recent paper by Cornelissen et al. (2013), who found that when people were asked to recall a behavior they had performed that had a moral or immoral outcome, they compensated in their dishonesty—that is, they were more likely to cheat on a subsequent task (Mazar et al., 2008). This moral compensation was explained by differences in participants' MSI, which were measured using the scale proposed in the current investigation. More specifically, they used our scale^2^ to demonstrate that the rise in MSI following a moral behavioral recall and the lowering of MSI following an immoral behavioral recall explained the magnitude of people's subsequent cheating behavior (i.e., more after moral behavior and less after immoral behavior). However, it is important to note that they did not demonstrate that (im)moral action recalls *changed* people's MSI from a baseline, a proposition that is central to our current theoretical argument. Our goal is to provide a theoretical foundation and empirically-driven examination of the MSI.

## The MSI and the self

We propose that the MSI resides in individuals' working self-concept, or current self-appraisal (Kernis and Johnson, 1990). The working self-concept is a malleable part of the self, which differentiates it from similar, more stable constructs, such as self-esteem (Rosenberg, 1965) and moral identity (Aquino and Reed, 2002). Like other areas of the working self-concept, people evaluate the state of their moral selves and attach either negative or positive labels to it based on cues from the social world and their own actions (Kernis and Goldman, 2003). Also like other parts of the working self-concept, the MSI is completely subjective, meaning that it is not a measure of the strength of one's moral judgments (Kohlberg, 1994), nor does it measure how moral (or immoral) a person actually is, but rather how moral (or immoral) she thinks she is. To take an extreme example, a devoutly religious person who dedicates his life to working with underprivileged children in the inner city might have a lower MSI following a spat with a fellow driver than a solipsistic investment banker who just made a small charitable donation following a similar argument. Though, individuals may vary in terms of how highly they value their moral selves, in general (see Aquino and Reed, 2002) people share a fairly universal desire to be moral (Dunning, 2007; Reed et al., 2007)—at least in terms of their self-perceptions of such morality (Mazar et al., 2008).

We define MSI as a person's malleable moral self-concept, that is, their self-concept related to the traits of the prototypically moral person (i.e., *caring, compassionate, helpful, hard-working, friendly, fair, generous, honest*, and *kind*)—derived from Aquino and Reed's (2002) work on the moral identity. While these nine traits are not expected to be an exhaustive representation of the traits of the moral prototype, we use these traits to evoke the mental representation of people's MSI. Below, we explain how the proposed construct of the MSI is associated with (and yet distinct from) other theoretically-related constructs.

### Moral identity

The MSI is distinct from moral identity in both its stability, as well as its implications for and responses to moral behaviors. Defined as “a self-conception organized around a set of moral traits” (Aquino and Reed, 2002, p. 1424), moral identity is comprised of an internalization subdimension, which is the importance to the self of possessing such traits, and a symbolization subdimension, which is the importance of demonstrating to others that one possesses those traits through one's behavior, style of dress, et cetera. Like the MSI, one's moral identity is conceptualized as a self-regulatory mechanism and is associated with various beliefs, attitudes, and behaviors (Aquino and Reed, 2002). But unlike the MSI, moral identity is a relatively stable trait (Aquino et al., 2009): “The definition of moral identity proposed here implies that if the identity is deeply linked to a person's self-conception, it tends to be relatively stable over time” (Aquino and Reed, 2002, p. 1425). If moral identity is highly regarded by the individual, it is predicted to lead to consistent moral actions throughout his or her life (Damon and Hart, 1992; Aquino and Reed, 2002). By contrast, the MSI is theorized to respond to events with a moral component, with a weak MSI stimulating moral action and a strong MSI allowing for moral relaxation (Cornelissen et al., 2013). Taken together, although the MSI is based on the moral traits identified by Aquino and Reed (2002), the MSI focuses on one's perception of how they are performing vis a vis these traits at a given moment, but not does not measure the extent to which a person values the moral traits (MI-internalization dimension) nor wishes to demonstrate them to others (MI-symbolization).

### Self-esteem

A vast amount of research exists on self-esteem (e.g., Deci and Ryan, 1995; Greenwald and Farnham, 2000; Crocker and Wolfe, 2001), which is defined as a person's global feelings of self-worth (Kernis and Goldman, 2003). Although, a person's self-esteem can change, it is unlikely to change in response to a single event or within a short period of time. Any instability in self-esteem usually occurs over an extended period (Rosenberg, 1986)—for example, from elementary to high school (e.g., McCarthy and Hoge, 1982). Distinctions have been made between global self-esteem and specific self-appraisals (similar to the MSI). Global self-esteem tends to be based on a generalized emotional response to the social world, whereas self-appraisals involve the cognitive appraisal of one's performance or acumen in some domain (Brown, 1993). While self-appraisals can influence self-esteem if they represent a core dimension of one's self-concept (Kernis et al., 1993; Pelham, 1995), global self-esteem and MSI differ from each other in three key ways: (1) self-esteem concerns a person's global feelings of self-worth rather than his or her specific moral self-appraisals, (2) self-esteem is relatively stable, and (3) self-esteem is more of an emotional than a cognitive response to the social world.

A more dynamic variation of self-esteem is state self-esteem, which is defined as a person's momentary assessment of self-regard. State self-esteem contains three sub-dimensions: performance (i.e., concern about one's abilities), social (i.e., concern about how others see oneself), and appearance (i.e., concern about how one physically appears; Heatherton and Polivy, 1991). Similar to our theorizing about the MSI, state self-esteem is affected by the environment. For example, Heatherton and Polivy (1991) find that state self-esteem decreases from a baseline following feedback about failure on an intellectual task and increases with interventions aimed at improving self-esteem. However, state self-esteem encompasses people's general feelings of self-worth rather than their specific feelings about their self-worth in the moral domain. Similar to the self-appraisal reasoning described above, we expect that while one's MSI would be likely to affect one's state self-esteem, the opposite is unlikely to be the case (i.e., one's general feeling of self-worth would not affect one's MSI).

### Actual, ought, and ideal selves

Self-discrepancy theory postulates that individuals have three “selves”: the actual self, or the person one is perceived to be, the ideal self, or the person one would like to be, and the ought self, or the person one should be (Higgins, 1987). These latter two selves are referred to as the “self-guides,” for they are thought to guide people's behavior and the nature of their self-assessments. Self-discrepancy theory contends that people are motivated to reach a state where their perceived actual self matches one of these self-guides. It also contends that discrepancies between what a person perceives to be his or her actual self and either the ideal or ought self lead to various types of negative emotions and discomfort. Despite apparent similarities, the construct of the MSI differs from self-discrepancy theory in two key ways. First, self-discrepancy theory concerns one's general self-assessment across domains rather than one's specific self-assessment in the moral domain (i.e., to measure one's self-discrepancy, people are asked to generate attributes related to each of the three selves). Second, self-discrepancy theory places significant importance on the source of the self view, proposing that each of these three selves are derived from either one's own self view *or* the individual's perception of how others perceive them (e.g., you have both the ought self that you perceive and an ought self that you think others perceive of you; see Higgins, 1987) and that the source of the self view is important because it affects the type of negative emotions or discomfort that results from discrepancies with the actual self. The MSI does not distinguish between the source of one's self-perceptions, as we contend that one's self-perceptions are a reflection of both one's own perceptions and the perceived perceptions of others. We also contend that the MSI is not exclusively derived from one of the two self-guides. Research on morality suggests that our moral standards come from a mix of the “oughts”—that is, the societally-dictated idea of what we should be (Kohlberg, 1971; Hoffman, 1975)—and “ideals,” that is, the what we (or others) would like ourselves to possess (Lapsley and Lasky, 2001; Monin and Jordan, 2009; Jordan et al., 2011). While we believe that the MSI comes from a combination of the ideal and ought selves and assert that both the ideal and ought are influential self guides, we argue that one's personal ideal self-standards are more relevant to the MSI than are standards derived from the surrounding social context. Supporting this assertion is fundamental research on identity, which suggests that the self-concept is derived from the actual and ideal selves (Wylie, 1974), as well as research on self-esteem (a construct that we theorize and demonstrate is related to the MSI) which has been empirically associated with actual-ideal discrepancies but not actual-ought discrepancies (Moretti and Higgins, 1990). We address the distinction between the MSI and self-discrepancy theory both theoretically and empirically in Study 2.

## The current research

Across five studies, we aim to formally present an instrument to measure the dynamics of people's moral selves, as well as to demonstrate its malleability in response to moral and immoral events. Study 1 demonstrates the convergent and discriminant validity of the MSI scale with other theoretically-related scales. Study 2 examines how the MSI is related to the *ideal* versus the *ought* self (Higgins, 1987). Studies 3 through 5 demonstrate the construct validity of the MSI, by demonstrating the malleability of this measure based on various events (i.e., feedback-related and self-related recalls). Study 3 looks at feedback related to social comparisons of moral behavior, and Study 4 examines feedback relative to one's own moral ideal. Lastly, Study 5 provides evidence that recall of one's moral or immoral behavior affects subsequent immoral behavior (Cornelissen et al., 2013), altering MSI in the predicted directions^3^.

## Study 1

Across two samples (1a and 1b), we compare our MSI measure with measures of theoretically-related constructs, including Moral Identity (Aquino and Reed, 2002), Generalized Self-esteem (Rosenberg, 1965), Moral Disengagement (Moore et al., 2012), Religiosity (Brown, 1962), Negative Reciprocity Norm (Eisenberger et al., 2004), and Sympathy (Ahmed and Jackson, 1979). We explain each of these constructs and their accompanying scales, as well as our hypothesized relationships with these constructs below.

### Moral self-image

We measured MSI by presenting nine traits perceived as prototypical of the ideally-moral person (Aquino and Reed, 2002). Using a nine-point Likert Scale (1 = *much less than the X person I want to be*; 9 = *much more than the X person I want to be*), we asked people to indicate where they were relative to their ideal self on each trait; see Supplementary Material.

### Moral identity

Moral identity is defined as having a self-conception organized around a set of moral traits. Moral identity possesses two dimensions, *internalization* and *symbolization*. Internalization is the importance people place on possessing these traits, and symbolism is the importance they place on demonstrating these traits to others (e.g., through membership in clubs or the clothes they wear). For example, an *internalization* item is, “It would make me feel good to be a person who has these characteristics,” whereas a *symbolization* item is, “I am actively involved in activities that communicate to others that I have these characteristics.” Using the Aquino and Reed (2002) 10-item scale (1 = *completely disagree*; 7 = *completely agree*), we measured both dimensions and had divergent predictions for each. Previous research has demonstrated that past moral actions affect the symbolic but not the internalized moral identity (Jordan et al., 2011) and that, instead, the internalized moral identity affects how people behaviorally respond to immoral events (Mulder and Aquino, 2013). Thus, we hypothesized that while one's MSI would not be affected by the importance one places on possessing moral traits (internalization), it would be (positively) affected by the extent to which one demonstrates the moral self to others (symbolization), as such public demonstrations would boost people's conceptions of their moral selves.

### Generalized self-esteem

Generalized self-esteem is defined as a person's global feelings of self-worth and -acceptance. We measured self-esteem using Rosenberg's (1965) 10-item measure. Items included, “On a whole, I am satisfied with myself,” and “At times, I think I am no good at all (reverse-scored)” (1 = *strongly disagree*; 5 = *strongly agree*). Although, self-esteem is considered a stable construct and MSI is considered a malleable construct, we predicted a positive relationship between MSI and generalized self-esteem, given that temporary self-appraisals have been found to be predictive of global self-esteem, particularly when these self-appraisals are a part of the self that the person considers central or core (see Kernis et al., 1993; Pelham, 1995).

### Moral disengagement

Moral disengagement is defined as, “an individual's propensity to evoke cognitions which restructure one's actions to appear less harmful, minimize one's understanding of responsibility for one's actions, or attenuate the perceptions of the distress one causes to others” (Moore, 2008, p. 129). In other words, moral disengagement is a person's ability to rationalize his or her immoral behavior in a way that helps reduce the negative feelings that would otherwise result. We measured moral disengagement using the eight-item Propensity to Morally Disengage Scale (Moore et al., 2012), which included, “People shouldn't be blamed for doing things that are technically wrong when all their friends are doing it too,” and “Some people have to be treated roughly because they lack feelings that can be hurt” (1 = *not at all*; 7 = *very much so*). We predicted that moral disengagement would be positively related to MSI because the more one is able to morally disengage from one's immoral actions, the greater one's MSI.

### Religiosity

Religiosity is defined as the extent to which a person holds various religious beliefs. We measured religiosity using the Other Orthodox Christian Beliefs subscale of Brown's (1962) Religiosity measure. Intuitively, religiosity may be related to the perception of oneself as moral; indeed, religiosity and people's desire to symbolize their moral self to others have been found to be positively correlated (Aquino and Reed, 2002). Thus, we hypothesized a positive relationship between religiosity and MSI.

### Negative reciprocity norm

The negative reciprocity norm is the belief that it is appropriate to retaliate against an immoral or unjust act leveled against oneself (Gouldner, 1960). We measured this construct using the nine-item scale of Eisenberger et al. (2004), which included, “If someone says something nasty to you, you should say something nasty back” and “If someone treats you badly, you should treat that person badly in return” (1 = *not at all*; 7 = *very much so*). The negative reciprocity norm has been found to be negatively related to the extent to which a person considers moral traits central to his or her self-concept (Aquino and Reed, 2002). As the MSI focuses on an assessment of the state of one's moral-self rather than how much one values a moral identity (which would likely be associated with someone's desire to retaliate for an act perceived as unjust), we did not expect any relationship between MSI and holding a norm of negative reciprocity.

### Sympathy

Sympathy is the ability to show concern for the needs and welfare of others (Eisenberg, 2000). We measured sympathy with the eight-item nurturance dimension of the Acceptance of Welfare scale (Ahmed and Jackson, 1979), which included, “Someone who is disabled will get my attention and aid” and “People in need deserve my sympathy and support” (1 = *strongly disagree*; 7 = *strongly agree*). Similar to the rationale behind our predictions for Negative Reciprocity Norm, we predicted a null relationship between MSI and Sympathy. The state of one's MSI should be unrelated to one's general beliefs about showing sympathy for those less fortunate.

### Positive and negative affect

In Sample 1b only we examined positive and negative affect because we wished to see how the state of the MSI related to individuals' affective states. We administered the PANAS (Watson et al., 1988), which presented participants with 10 positive (e.g., *proud, active*) and 10 negative (e.g., *upset, nervous*) items and asked them to rate themselves on each item based on how they were feeling at the current moment (1 = *not at all*; 7 = *very much so*). As the PANAS measures a state-based construct (similar to the MSI), and because people's moral selves are an integral part of their self-concepts, we predicted that the MSI would be positively related to positive affect and negatively related to negative affect.

### Gender and age

While there is some evidence that women reason differently (Jaffee and Hyde, 2000) and perhaps more complexly about moral issues than men (Wark and Krebs, 1996; White, 1999), there is no evidence to suggest that women think of themselves as any more or less moral then men. Similarly, there is evidence that moral behavior changes from adolescence into adulthood but is fairly stable in adulthood (Eisenberg et al., 2005), the age category of our samples. Thus, we predicted null relationships between MSI and both the demographic variables of age and gender.

### Participants—sample 1a

Participants were 574 American adults from a Mechanical Turk (Mturk) sample (*M*_age_ = 32.89, *SD* = 11.04, 48% female). They were invited to take part in a 20-min study in exchange for $0.55. Thirty participants did not pass the attention checks and thus were eliminated from the analyses, leaving a final sample of 544 on which to run the analyses.

### Participants—sample 1b

Participants were 515 American adults from an Mturk sample (*M*_age_ = 31.88, *SD* = 8.57, 49% female). They were invited to take part in a 20-min study in exchange for $0.60. Sixteen participants did not pass the attention checks and thus were eliminated from the analyses, leaving a final sample of 499 on which to run the analyses.

### Procedures

Participants read a consent form and, if they agreed to the terms, logged on to the study website. They completed all measures (with the NRN and Sympathy scales only in Sample 1a and the PANAs only in Sample 1b) stated above in a randomized order; however, either the MSI scale or the moral identity scale always came first.

### Results and discussion

All results (including Cronbach Alphas for the measures) are contained in Tables 1, 2.

**Table 1:** **Study 1 Sample 1a—Scale intercorrelations and reliabilities**.

	**MSI**	**MIs**	**MIi**	**GSE**	**MD**	**RELIG**	**NRN**	**SYMP**	**Age**	**Sex**
MSI	0.88									
MIs	0.26^***^	0.83								
MIi	0.03	0.29^***^	0.84							
GSE	0.20^***^	0.17^***^	0.26^***^	0.93						
MD	0.15^***^	0.001	−0.44^***^	−0.18^***^	0.84					
RELIG	0.17^***^	0.25^***^	0.14^***^	0.11^**^	0.009	0.87				
NRN	0.05	−0.19^***^	−0.37^**^	−0.14^***^	0.55^***^	−0.09^*^	0.86			
SYMP	0.03	0.26^***^	0.63^**^	0.26^***^	−0.51^***^	0.12^**^	−0.48^***^	0.95		
Age	0.09^*^	−0.02	0.16^***^	0.21^***^	−0.22^***^	0.14^***^	−0.12^**^	0.13^**^	−	
Sex	−0.06^†^	−0.11^**^	−0.23^***^	−0.02	0.25^***^	−0.13^**^	0.19^***^	−0.17^***^	−0.17^***^	−

*Cronbach alphas contained in the diagonals*.

† p < 0.10;

* p ≤ 0.05;

** p ≤ 0.01;

*** *p ≤ 0.001*.

*MSI, moral self-image; MIs, moral identity–symbolization; MIi, moral identity–internalization; GSE, generalized self-esteem; MD, moral disengagement; RELIG, religiosity; NRN, negative reciprocity norm; SYMP, sympathy. For gender, 1, female; 2, male*.

**Table 2:** **Study 1 Sample 1b—Scale intercorrelations and reliabilities**.

	**MSI**	**MIs**	**MIi**	**GSE**	**MD**	**RELIG**	**PANAS-P**	**PANAS-N**	**Age**	**Gender**
MSI	0.88									
MIs	0.23^***^	0.85								
MIi	−0.07	0.38^***^	0.82							
GSE	0.31^***^	0.23^***^	0.22^***^	0.94						
MD	0.15^***^	−0.05	−0.46^***^	−0.16^**^	0.84					
RELIG	0.14^**^	0.30^***^	0.25^***^	0.13^**^	−0.03	0.90				
PANAS−P	0.32^***^	0.36^***^	0.17^***^	0.50^***^	0.02	0.27^***^	0.92			
PANAS−N	−0.01	−0.08^†^	−0.31^***^	−0.39^***^	0.32^***^	−0.02	−0.04	0.92		
Age	0.06	0.00	0.11^*^	0.18^***^	−0.20^***^	0.14^**^	0.16^***^	−0.13^**^	−	
Gender	0.09^*^	−0.10^*^	−0.22^***^	0.04	0.18^***^	−0.14^**^	0.03	0.05	−0.14^**^	−

*Cronbach alphas contained in the diagonals*.

† p < 0.10;

* p ≤ 0.05;

** p ≤ 0.01;

*** *p ≤ 0.001*.

*MSI, moral self-image; MIs, moral identity–symbolization; MIi, moral identity–internalization; GSE, generalized self-esteem; MD, moral disengagement; RELIG, religiosity; PANAS-P, PANAS positive affect; PANAS-N, PANAS negative affect. For gender, 1, female; 2, male*.

Demonstrating convergent validity, across both samples, MSI was positively related to symbolic (but not internalized) moral identity, generalized self-esteem, moral disengagement, and religiosity; also as predicted, demonstrating divergent validity, we found no relationship between MSI and negative reciprocity norms and sympathy. However, in contrast to predictions, we found that in Sample 1a (but not 1b) age was positively related to MSI, with older individuals having higher MSIs than younger individuals. In Sample 1a gender was marginally negatively related to MSI, with women reporting higher MSIs than men; however in Sample 1b the directionality of this relationship flipped such that men reported higher MSIs than women.

We then conducted an exploratory factor analysis to explore the factor structure of our MSI scale, predicting that a single factor would emerge from the data. We conducted a principal components analysis with an oblique rotation method, which would allow for the potential factors to be correlated with one another (*direct oblimin*). From an inspection of the scree plot, eigenvalues, and factor loadings across both samples, only one factor (Sample 1a: Eigenvalue = 4.48; Sample 1b: Eigenvalue = 4.68) emerged from the data. This factor explained between 51.96% (1a) and 52.37% (1b) of the variance, and all items loaded on to this factor at a loading of 0.53 (*How hardworking are you relative to your ideal?*) or higher^4^. In sum, across Samples 1a and 1b, we found that MSI was positively related to symbolic moral identity, generalized self-esteem, and religiosity and negatively related to moral disengagement. We also found that our scale contained a single factor structure, which explained at least 50% of the variance across both studies. These findings provide suggestive evidence of the validity of MSI as a unique construct.

## Study 2

In Study 2, we examine how the MSI is related to the ideal versus ought self (Higgins, 1987). As noted earlier in the Introduction, self-discrepancy theory postulates that individuals have the actual self and two “self-guides,” including the ought self and the ideal self (Higgins, 1987). Self-discrepancy theory argues that people are motivated to align their perceived actual self with one of these self-guides and that discrepancies between the actual self and either the *ought* or *ideal* self lead to negative emotions and discomfort. As argued above, we propose that the MSI is primarily comprised of one's perceived moral self relative to one's own moral ideal self-standard rather than relative to an externally-imposed standard (i.e., the ought). That is, the MSI assesses who a person perceives him or herself to be relative to the ideal moral person that he or she wishes to be—not the moral person he or she thinks *others* wish him or herself to be. This contention is derived from research demonstrating that moral or immoral behaviors do not need to be witnessed by others in order to elicit compensatory effects; only the individual him or herself needs to be aware of the event (Sachdeva et al., 2009; Jordan et al., 2011), as well as research suggesting that the self concept is comprised of a mix of actual and ideal states (Wylie, 1974).

In order to empirically test this idea, half of the participants in the current study completed the MSI scale as it was originally written (i.e., in a way that measured the ideal moral self). The other half of participants completed a version of the scale in which we asked people not about the moral self they perceived themselves to possess relative to where they wanted to be (*ideal*) but rather about the moral self they perceived themselves to possess relative to what they thought *others* wanted them to possess (*ought*). Along with one of these two versions of the scale, participants also completed the same measures administered to Sample 1b in Study 1.

Although, we contend that the *ought* self is relevant for the MSI, we predicted that the ideal MSI would be a better fit than the *ought* moral self with our proposed model.

### Participants, design, and procedures

Participants were 590 American adults from an Mturk sample (*M*_age_ = 35.94, *SD* = 11.34, 50.5% female). Participants were invited to take part in a 15-min study in exchange for $0.75 compensation.

Participants were randomly assigned to complete either the MSI scale or a version of the MSI scale in which we asked about their *ought* moral selves. Specifically, in the *ought self* condition, instead of asking participants to indicate how *caring, compassionate, fair*, et cetera he or she was at the present time relative to the person who he or she wanted to be, we phrased these items so that the participant was asked to indicate how *caring, compassionate, fair*, et cetera he or she was at the present time relative to who *others* wanted him or her to be. All participants then completed measures of moral identity (Aquino and Reed, 2002), generalized self-esteem (Rosenberg, 1965), moral disengagement (Moore, 2008; Moore et al., 2012), religiosity (Brown, 1962), and positive and negative affect (Watson et al., 1988).

### Results and discussion

All results (including Cronbach Alphas for the measures) are contained in Tables 3, 4.

**Table 3:** **Study 2—Scale intercorrelations and reliabilities for the *Ideal* moral self**.

	**MSI**	**MIs**	**MIi**	**GSE**	**MD**	**RELIG**	**PANAS-P**	**PANAS-N**	**Age**	**Gender**
MSI	0.88									
MIs	0.34^***^	0.84								
MIi	0.05	0.36^***^	0.85							
GSE	0.20^***^	0.21^***^	0.15^**^	0.93						
MD	0.14^*^	−0.02	−0.46^***^	−0.11^†^	0.81					
RELIG	0.17^**^	0.30^***^	0.30^***^	0.08	−0.10^†^	0.89				
PANAS−P	0.33^***^	0.30^***^	0.25^***^	0.42^***^	−0.01	0.24^***^	0.92			
PANAS−N	−0.03	0.05	−0.16^**^	−0.26^***^	0.31^***^	0.02	−0.11	0.88		
Age	−0.03	0.004	0.15^**^	0.08^***^	−0.19^***^	0.20^***^	0.15^**^	−0.08	−	
Gender	−0.03	−0.21^***^	−0.18^**^	−0.06	0.20^***^	−0.18^**^	0.002	−0.03	−0.08	−

*Cronbach alphas contained in the diagonals*.

† p < 0.10;

* p ≤ 0.05;

** p ≤ 0.01;

*** *p ≤ 0.001*.

*MSI, moral self-image; MIs, moral identity–symbolization; MIi, moral identity–internalization; GSE, generalized self-esteem; MD, moral disengagement; RELIG, religiosity; PANAS-P, PANAS positive affect; PANAS-N, PANAS negative affect. For gender, 1, female; 2, male*.

**Table 4:** **Study 2—Scale intercorrelations and reliabilities for the *Ought* moral self**.

	**MSI**	**MIs**	**MIi**	**GSE**	**MD**	**RELIG**	**PANAS-P**	**PANAS-N**	**Age**	**Gender**
MSI	0.91									
MIs	0.42^***^	0.87								
MIi	0.41^***^	0.33^***^	0.87							
GSE	0.40^***^	0.26^***^	0.25^***^	0.94						
MD	−**0.10**	0.05	−0.48^***^	−0.24^***^	0.84					
RELIG	0.22^***^	0.23^***^	0.018^**^	0.11^†^	0.04	0.89				
PANAS−P	0.39^***^	0.37^***^	0.21^***^	0.42^***^	0.03	0.22^***^	0.93			
PANAS−N	−0.16^**^	−0.06	−0.38^***^	−0.36^***^	0.42^***^	0.01	0.02	0.90		
Age	0.08	−0.04	0.14^**^	0.04	−0.16^**^	0.14^**^	0.05	−0.10	−	
Gender	−0.19^**^	−0.18^**^	−0.30^***^	−0.09	0.23^***^	−0.30^***^	−0.05	0.08	−0.13^*^	−

*Cronbach alphas contained in the diagonals. Correlations that are bolded are those in which the relationship differed between the ought and the ideal moral self*.

† p < 0.10;

* p ≤ 0.05;

** p ≤ 0.01;

*** *p ≤ 0.001*.

*MSI, moral self-image; MIs, moral identity–symbolization; MIi, moral identity–internalization; GSE, generalized self-esteem; MD, moral disengagement; RELIG, religiosity; PANAS-P, PANAS positive affect; PANAS-N, PANAS negative affect. For gender, 1, female; 2, male*.

For the MSI (i.e., the *ideal* moral self), all relationships found in Study 1 (except for the relationship with gender) were replicated in the current study. Specifically, demonstrating the convergent validity of the proposed construct, MSI was positively related to symbolic (but not internalized) moral identity, generalized self-esteem, moral disengagement, religiosity, and positive affect. And again, demonstrating the divergent validity of the MSI with other constructs, we found no relationship between the MSI and negative affect or age. In this study, there was no relationship with gender.

In contrast, while several of the relationships found between MSI and the other explored constructs were replicated when using the *ought* version of the MSI scale, unlike the *ideal* MSI, the *ought* version showed a moderate positive correlation with the internalization subdimension of moral identity (Aquino and Reed, 2002), no correlation with moral disengagement (Moore, 2008), and a positive correlation with generalized self-esteem (Rosenberg, 1965), which was double the magnitude as witnessed for the *ideal* moral self. We also saw a moderate-sized correlation with gender, such that women reported having a greater moral self as perceived by others.

Thus, it appears that except for the negative correlation with negative affect, the *ideal* MSI more accurately captured our hypothesized relationships with the predicted related (and unrelated) constructs. To empirically test this assertion, we used the confirmatory factor analysis function of LISREL 8.80 maximum likelihood estimation method to compare our purported model using both the *ideal* MSI and the *ought* moral self via assessing the chi-square differences between the two models. As done in the previous studies, model fit was assessed by the Root Mean Square Error of Approximation (RMSEA), the Normed-fit Index (NFI), and the Comparative Fit Index (CFI).

The first model tested was the purported model in which the (*ideal*) MSI, the symbolization and internalization sub-dimensions of moral identity, generalized self-esteem, moral disengagement, religiosity, and positive and negative affect were analyzed as distinct factors. When using the *ideal* MSI, this eight-factor model had a good fit with the data, χ^2^(1801) = 3917.94; RMSEA = 0.064 (0.061, 0.066); NFI = 0.87; CFI = 0.93. In contrast, while the model using the *ought* MSI also showed sufficient model fit, χ^2^(1801) = 3898.68; RMSEA = 0.063 (0.060, 0.065); NFI = 0.90; CFI = 0.95, it was inferior to the one using the *ideal* MSI, Δχ^2^(1) = 19.26, *p* = 0.00001^5^.

In sum, while we did find that the *ought* moral self showed many of the same relationships as were found with the *ideal* MSI, the *ought* moral self was strongly positively correlated with individuals' internalized moral identity, which is the more “trait-like,” stable dimension of the two moral identity subdimensions (Jordan et al., 2011). It was also strongly positively correlated with the generalized self-esteem—a stable personality dimension. Taken together, it appears that the *ought* moral self mimics more of a stable, individual difference than does the *ideal* MSI. As stated earlier, we see the MSI not as being a stable, individual difference but as a state that responds to people's moral actions and social comparisons to the world around them. More research is required to make statements about the stability of the *ought* moral self with confidence.

Unlike the *ideal* MSI, the *ought* moral self was negatively correlated with negative affect. This is a relationship that was predicted for the *ideal* moral self but not witnessed in either Studies 1b or Study 2. Why this is is unknown. Perhaps, as highly social beings (Aronson, 2003), thinking about how others see you more strongly elicits negative emotions than does thinking about one's own moral self-evaluation. And lastly, we found that the *ought* moral self was higher for females than for males. While this was not hypothesized (nor found) for the *ideal* MSI, it was found for the *ideal* moral self in Study 1's Sample 1a. Why it was not found in the current study is unknown. However, it is unsurprising that women reported having a higher moral self as conceived by others in their social world, as there is evidence that society views women as being more moral and virtuous than men (Fiske et al., 1999, 2002; White, 1999).

In Studies 3, 4, and 5 we examine a fundamental assertion that underlies our theorizing about MSI, namely that the MSI responds to explicit feedback about one's moral state relative to others and to one's own moral ideal. Research on the self demonstrates that the self-concept is influenced by three primary sources originating in the social environment: social comparison, feedback, and an individual's actions (Kernis and Goldman, 2003). In Study 3, we explore the effect of the first of these three sources, social comparison, on changes to a person's MSI (looking specifically at feedback in Study 4 and actions in Study 5). In Studies 4 and 5, in order to examine the independent contribution of MSI, we then investigate whether such feedback influences related constructs. Specifically, we investigate whether such feedback not only affects the MSI but also moral identity (Aquino and Reed, 2002), generalized self-esteem (Rosenberg, 1965), and state self-esteem (Heatherton and Polivy, 1991).

## Study 3

In Study 3, we explore one of the primary sources of influence originating in the social environment (Kernis and Goldman, 2003), social comparison information, and examine how it influences the MSI. We predicted that the MSI would be affected by this feedback with positive feedback leading to an increase in one's MSI and negative feedback leading to a decrease.

### Participants and design

Participants were 59 international business students (56% women, *M*_age_ = 21.86, *SD* = 2.45) at a university in the Netherlands who participated in exchange for €6. We presented all materials in English and randomly assigned participants to one of two moral-valence conditions: above average moral or below average moral. Thirteen participants were excluded from the analyses, leaving us with a total of 46 participants on which to run the analyses^6^.

### Procedures

Participants were required to complete our MSI scale at least 15 h prior to coming into the lab. We sent them a link to the MSI scale immediately after they had signed up for the study.

When the participants arrived in the lab, we told them they would be completing a study about their environmental conservation behavior. Participants were required to write a short essay about, “what actions you take in support of environmental conservation and why you think these are important.” We used the topic of environmental conservation behavior because previous research has found this topic to be related to people's moral selves (Mazar and Zhong, 2010). We told each participant that the experimenter would interrupt him or her after several minutes to obtain more information about the essay he or she had just written. Before the experimenter came in, the computer delivered a message to the participant. They were told that the essay they had written was actually part of a standardized measure of people's “MIP,” or “how much moral traits are a part of your identity and who you are.” We said that the measure assessed both the vocabulary they used and the speed at which they typed to generate a score that we could compare with the scores of others in the population. The experimenter then opened the door and gave participants a sheet that further explained this measure and the ranges of scores that were possible; these ranges were presented in five categories ranging from a very low moral self-identity to a very high one. The experimenter told participants that he would type a personal code into the main computer that would allow the participant to see his or her score. He assured each participant that this score would only be visible to the participant. Following this interaction, the participant saw his or her score. This score fell into one of two categories: very high or very low relative to the rest of the population. Each score was accompanied by the percentiles of the population in which they fell (e.g., 1st–11th percentile; 88–99th percentile), hence providing a point of social comparison. The participant then completed the MSI scale once again.

Before leaving the lab, we asked participants to indicate the range their score fell into from a choice of five options. Finally, they were fully debriefed, a process that included telling them that the measure and associated feedback were completely bogus.

### Results

#### Manipulation checks

All participants selected the correct score range on the manipulation check.

#### MSI

In order to analyze our hypothesis that feedback would be directly related to a change in individuals' MSI, we subtracted their score on the pre-test from their score on the post-test (for similar methods, see Heatherton and Polivy, 1991). In a case like this, where a change score is used as the dependent, rather than independent, variable, polynomial regression is not necessary nor appropriate (see Edwards, 2002)^7^.

Participants in both the extremely positive (*M* = −0.25, *SD* = 0.76) and extremely negative (*M* = 0.22, *SD* = 1.19) conditions began with equivalent MSIs, *F*_(1, 44)_ = 2.65, *p* = 0.11. While the post-test scores between the extremely positive (*M* = 0.01, *SD* = 0.91) and extremely negative (*M* = 0.10, *SD* = 1.18) conditions also did not differ by condition, *F*_(1, 44)_ = 0.84, *p* = 0.77, the change between the pre- and post-test did differ by condition, *F*_(1, 44)_ = 4.35, *p* = 0.04, η^2^ = 0.09. Specifically, those who received extremely positive feedback about the states of their moral selves showed an increase between scores on the pre- and post-test (*M* = 0.25, *SD* = 0.70), whereas those who received extremely negative feedback about the states of their moral selves showed a decrease between scores on the pre- and post-test (*M* = −0.13, *SD* = 0.48).

### Discussion

As predicted, we found that feedback regarding people's moral selves relative to others led to self-reported changes in their MSI. People who were told they had a moral self that was extremely above average had a positive change between pre- and post-testing, whereas those who were told that they had a moral self that was extremely below average showed a negative change. We wish to acknowledge that while the difference between the two conditions for the pre-test scores was not significant, the extremely positive condition did start at a lower point than the extremely negative condition. This lower pre-test score increased the chances that a mere regression to the mean would produce MSI change scores that would increase for the former condition more so than for the latter. In order to explore the robustness of this effect more thoroughly, in the following two studies, we examine the effects of two additional sources of self-image impact on people's MSI.

## Study 4

In Study 4, we explore the effect of the second of the three sources of impact to one's self-concept (Kernis and Goldman, 2003), feedback, on changes to a person's MSI (Kernis and Johnson, 1990). Specifically, we examine how explicit feedback about the state of one's moral self relative to one's own personal ideal influences the MSI in both positive and negative ways. To continue the investigation of discriminant validity, we also examined the change in MSI relative to the change in other potentially-related constructs. Specifically, consistent with our argument that feedback about the moral self will only lead to changes to the MSI, we also asked people to assess themselves on four amoral traits (i.e., *sporty, organized, smart, and sociable*). To rule out the possibility that our moral feedback changed people's general self-concept (rather than specifically their MSI), we also examined how our feedback changed people's generalized (Rosenberg, 1965) and state self-esteem (Heatherton and Polivy, 1991). To investigate whether such feedback affected other dimensions of the moral self, we also examined changes to their moral identity (Aquino and Reed, 2002). We predicted that changes following this feedback would only occur on one's MSI and not on the amoral control traits, self-esteem, or moral identity.

### Participants

Participants were 130 international business students (52% female, *M*_age_ = 21.06, *SD* = 3.03) at a university in The Netherlands who participated in exchange for €4. We presented all materials in English. Fifteen participants were excluded from the analyses^8^, leaving us with a working total of 115 participants.

### Design and procedures

We had three feedback conditions: *meeting ideal moral self*, *almost meeting ideal moral self*, and *a ways away from meeting the ideal moral self*. We chose these types of feedback because they represented people's achievement of their ideal moral self in addition to being both close and far from this ideal state.

Participants were required to sign up for the study at least 20 h ahead of their scheduled session. Immediately upon signing up, we sent them a link to an online data collection site, where they completed the pre-test measures: MSI, control trait ratings, moral identity, generalized self-esteem, and state self-esteem. They had to complete these measures at least 15 h in advance of their session in order to participate in the laboratory portion of the study.

Participants came into the lab at least 15 h after completing the pre-test, ostensibly for a study on “e-tests.” They were first asked a series of questions about their prosocial behavior and asked to write an essay about what they do to help other people in their daily lives. As in Study 2, we then told them that what they actually just took was a measure called the “MIP,” which along with the questions they answered online prior to coming into the lab, indicates how close they are to meeting “the moral self they ideally wish to be.” We then gave them both verbal and graphic feedback about where they fell on this scale. Specifically, participants were told that they *met the moral self that they aspire to be, have almost met the moral self that they aspire to be*, or were *a ways away from meeting the moral self they aspire to be*, depending on their randomly-assigned condition. We accompanied this feedback with diagrams to show how close they were to their ideal moral self.

Participants then took all the pre-test measures a second time and then were fully debriefed, which included being told that the measure and associated feedback was completely bogus.

### Results

All pre- and post-test correlations are contained in Table 5. The correlations for the pre-test replicated those found in Study 1 (Samples 1a and 1b) and Study 2 for both the symbolic (positive) and internalized (none) moral identity scores. They also replicated the results found for Study 1 (Sample 1a) for both gender (positive) and age (marginally negative). However, surprisingly, the pre-test MSI was not correlated with generalized self-esteem, as found in the previous studies.

**Table 5:** **Study 4—Pre- and Post-test scale intercorrelations and reliabilities**.

	**MSI_1_**	**MIs_1_**	**MIi_1_**	**GSE_1_**	**SSE_1_**	**MSI_2_**	**MIs_2_**	**MIi_2_**	**GSE_2_**	**SSE_2_**	**Age**	**Gender**
MSI_1_	0.78											
MIs_1_	0.29^***^	0.75										
MIi_1_	0.14	0.47^***^	0.75									
GSE_1_	−0.03	−0.07	0.004	0.85								
SSE_1_	−0.02	−0.02	−0.08	0.70^***^	0.86							
MSI_2_	0.83^***^	0.24^**^	0.06	0.08	0.04	0.89						
MIs_2_	0.27^**^	0.80^***^	0.42^***^	−0.02	−0.05	0.22^*^	0.82					
MIi_2_	0.11	0.44^***^	0.75^***^	−0.07	−0.16	0.03	0.50^***^	0.82				
GSE_2_	−0.02	−0.02	−0.06	0.86^***^	0.71^***^	0.02	0.04	−0.01	0.87			
SSE_2_	−0.06	−0.04	−0.06	0.68^***^	0.89^***^	−0.01	−0.03	−0.11	0.75^***^	0.88		
Age	0.19^*^	0.05	−0.02	−0.02	0.02	0.20^*^	−0.02	0.02	−0.11	0.00	−	
Gender	−0.17^†^	−0.13	−0.15	0.28^***^	0.17^†^	−0.13	−0.10	−0.13	0.27^**^	0.18^*^	0.04	−

*Cronbach alphas contained in the diagonals*.

† p < 0.10;

* p ≤ 0.05;

** p ≤ 0.01;

*** *p ≤ 0.001*.

*MSI, moral self-image; MIs, moral identity–symbolization; MIi, moral identity–internalization; GSE, generalized self-esteem; SSE, state self-esteem. _1_indicates that it was taken in the pre-test. _2_indicates that it was taken in the post-test. For gender, 1, female; 2, male*.

As can be seen in Table 6, the MSI changed in the predicted directions based on the feedback we provided, with the *met* feedback raising people's MSI between pre- and post-test and the *a ways away* feedback lowering people's MSI, *F*_(2, 112)_ = 3.33, *p* = 0.04, η^2^ = 0.06. However, this was not the case for symbolic moral identity, *F*_(2, 112)_ = 2.20, *p* = 0.12, generalized self-esteem, *F*_(2, 112)_ = 0.01, *p* = 0.99, state self-esteem, *F*_(2, 112)_ = 1.41, *p* = 0.25, and the amoral traits, *F*_(2, 112)_ = 2.19, *p* = 0.12. And counter to our predictions, our feedback affected internalized moral identity, *F*_(2, 112)_ = 3.52, *p* = 0.03, η^2^ = 0.06, with those receiving the *almost met* feedback showing an increase from pre- to post-test and the *met* condition showing a decrease. There was no significant difference between either of these two conditions and the *a ways away* condition.

**Table 6:** **Study 4—Pre- and Post-test scale means and change scores**.

**Measure**	**Pre-test mean (SD)**	**Post-test mean (SD)**	***Met* Condition mean (SD)**	***Almost Met* Condition mean (SD)**	***A ways away* Condition mean (SD)**
MSI	5.06 (0.80)	5.01 (0.90)	0.11_a_ (0.46)	−0.07_a, b_ (0.51)	−0.18_b_ (0.51)
MIs	5.74 (0.83)	5.77 (0.83)	−0.20 (0.60)	−0.10 (0.66)	0.09 (0.64)
MIi	4.14 (1.02)	4.07 (1.03)	−0.14_a_ (0.59)	0.21_b_ (0.66)	0.02_a, b_ (0.47)
GSE	3.78 (0.57)	3.76 (0.56)	−0.02 (0.26)	−0.01 (0.27)	−0.01 (0.37)
SSE	3.40 (0.49)	3.47 (0.50)	0.03 (0.26)	0.12 (0.22)	0.05 (0.22)
Control traits	4.52 (0.98)	4.53 (0.90)	0.04 (0.57)	−0.12 (0.51)	0.12 (0.43)

*MSI, moral self-image; MIs, moral identity–symbolization; MIi, moral identity–internalization; GSE, generalized self-esteem; SSE, state self-esteem. For those variables with a significant omnibus ANOVA, means with different subscripts significantly differ at a p < 0.05*.

### Discussion

As we predicted, telling people that they had achieved their ideal moral selves led them to increase their MSI, whereas telling them that they were *a ways away* from achieving their ideal moral selves led them to decrease their MSI. This feedback did not affect people's ratings on the amoral traits, their general or state self-esteem (which is in contrast to previous results, see Barkan et al., 2012), nor their symbolic moral identity.

However, it did affect their internalized moral identity, an unexpected finding both because internalized moral identity is argued to be a stable trait (Aquino and Reed, 2002) and because it has been found to be so in other research (e.g., Jordan et al., 2011). We therefore did not predict that our feedback would change ratings on this construct, which represents the importance that people place on possessing moral traits. Participants placed more importance on possessing moral traits when we told them that they had almost reached their ideal moral selves than when we told them that they had met their ideal moral selves. These results could have been due to an aspiration-level phenomenon (Zhang et al., 2007). In other words, people may have lowered the importance of moral traits when they believed they had met the goal and may have raised the importance when they were told that they were “almost there.” Future, research on the variance of internalized moral identity should investigate this possibility.

As discussed earlier, in addition to feedback, people's self-concepts are influenced by their own actions (Kernis and Goldman, 2003). As such, people's MSI should also respond to their moral actions and to their recalls about their moral actions (Sachdeva et al., 2009; Jordan et al., 2011). Thus, the purpose of Study 5 is to examine how recall of moral and immoral behavior changes people's MSI.

## Study 5

Study 5 aimed to explore the third source of influence on one's self concept—an individual's actions (Kernis and Goldman, 2003)—by analyzing whether the recall of one's (im)moral actions alters one's MSI. Cornelissen et al. (2013) used the current MSI scale to demonstrate that the effects of recalling one's (im)moral actions on future immoral behavior can be explained by the state of one's MSI. Specifically, they asked people to recall a time when they acted in a way that intentionally harmed another person (immoral) or intentionally benefitted another person (moral). They then had them engage in a task where they could cheat for their own personal gain (adapted from Mazar et al., 2008) and found that people who recalled harming another person cheated on fewer tasks than those who recalled helping another person and that these compensatory effects were explained by the level of a person's MSI, as measured by the current scale^9^. This finding would suggest that the change to one's MSI caused by (im)moral actions explains people's subsequent moral compensation behavior.

However, and as noted before, although these authors used the current scale to demonstrate this mediation, they did not demonstrate that people's MSI actually changed from a baseline; they also did not compare that effect to other possible changes in similarly related constructs^10^. Thus, in Study 4, we used these exact recalls to examine how they changed people's MSI from a baseline level. We also included a control condition to examine the directionality of the effects. We predicted that whereas recalling an immoral action would lower people's MSI, recalling a moral action would raise people's MSI.

### Participants

Participants were 119 international business students (48% female, *M*_age_ = 21.68, *SD* = 2.96) at a university in The Netherlands who participated in exchange for €4. All materials were presented in English. We excluded 12 people, leaving us with a working total of 107 participants^11^.

### Design and procedures

We had two conditions that were identical to those used by Cornelissen et al. (2013): recalling an intentional action one engaged in that harmed another person or recalling an intentional action one engaged in that benefitted another person. For example, in the unethical condition, participants wrote about behaviors such as borrowing money from another person and then waiting until the other person likely had forgotten so that he/she did not have to pay the person back, or delivering low-quality work on a group project in the expectation that other members would compensate for it. In the ethical condition, participants wrote about behaviors such as loaning a friend money that one had set aside for new clothes or joining a friend for an event that the other person did not feel comfortable attending alone despite being tired. We also included a control condition in which participants were asked to recall their last visit to the grocery store.

All other procedures were identical to those used in Study 4: participants were required to complete the pre-test measures (i.e., MSI, control traits, moral identity, state self-esteem, and generalized self-esteem) at least 15 h in advance of their session in order to participate in the laboratory portion of the study. In the laboratory session, they completed the recall task and then all pre-test measures once again. Finally, they were fully debriefed.

### Results

All pre- and post-test correlations are included in Table 7. The correlations for the pre-test replicated those found in S1 (Samples 1a and 1b), Study 2, and Study 4 for both the symbolic (positive) and internalized (none) moral identity scores. They, however, failed to show any effects for either gender or age. And as found in Study 4 (albeit not Study 1 or 2), the pre-test MSI was not correlated with generalized self-esteem. We discuss possible reasons for this in the General Discussion.

**Table 7:** **Study 5—Pre- and Post-test scale intercorrelations and reliabilities**.

	**MSI_1_**	**MIs_1_**	**MIi_1_**	**GSE_1_**	**SSE_1_**	**MSI_2_**	**MIs_2_**	**MIi_2_**	**GSE_2_**	**SSE_2_**	**Age**	**Gender**
MSI_1_	0.72											
MIs_1_	0.32^***^	0.64										
MIi_1_	0.12	0.46^***^	0.72									
GSE_1_	0.09	0.04	−0.04	0.80								
SSE_1_	−0.04	−0.02	−0.12	0.73^***^	0.86							
MSI_2_	0.75^***^	0.35^***^	0.25^**^	0.02	−0.03	0.79						
MIs_2_	0.21^**^	0.78^***^	0.49^***^	−0.06	−0.09	0.29^**^	0.76					
MIi_2_	0.15	0.43^***^	0.78^***^	−0.07	−0.14	0.24^*^	0.48^***^	0.78				
GSE_2_	0.00	−0.02	−0.02	0.86^***^	0.73^***^	0.04	−0.07	0.03	0.78			
SSE_2_	−0.01	−0.03	−0.03	0.68^***^	0.84^***^	−0.02	−0.07	−0.06	0.70^***^	0.84		
Age	0.01	−0.01	−0.02	−0.09	−0.07	0.02	0.08	0.08	−0.10	−0.09	−	
Gender	−0.02	0.04	−0.10	0.05	0.08	0.02	−0.02	−0.06	0.15	0.05	−0.01	−

*Cronbach alphas contained in the diagonals*.

* p ≤ 0.05;

** p ≤ 0.01;

*** *p ≤ 0.001*.

*MSI, moral self-image; MIs, moral identity–symbolization; MIi, moral identity–internalization; GSE, generalized self-esteem; SSE, state self-esteem. _1_indicates that it was taken in the pre-test. _2_indicates that it was taken in the post-test. For gender, 1, female; 2, male*.

As can be seen in Table 8, the MSI changed in the predicted directions based on people's recalled situations, *F*_(2, 103)_ = 3.79, *p* = 0.03, η^2^ = 0.07: recalls of people's moral actions led to increases in their MSI, recalls of people's immoral actions led to decreases in their MSI, and the control recall led to virtually no change. However, this was not the case for the moral identity measure [both symbolic, *F*_(2, 103)_ = 0.20, *p* = 0.70, and internalized, *F*_(2, 103)_ = 0.46, *p* = 0.64], generalized self-esteem, *F*_(2, 103)_ = 0.31, *p* = 0.73, state self-esteem, *F*_(2, 103)_ = 0.48, *p* = 0.62, and the control traits, *F*_(2, 103)_ = 2.24, *p* = 0.11.

**Table 8:** **Study 5—Pre- and Post-test scale means and change scores**.

**Measure**	**Pre-test mean (SD)**	**Post-test mean (SD)**	***Moral* Condition mean (SD)**	***Immoral* Condition mean (SD)**	**Control condition mean (SD)**
MSI	5.06 (0.73)	5.05 (0.76)	0.11_a_ (0.40)	−0.21_b_ (0.59)	−0.02_a, b_ (0.55)
MIs	4.13 (0.94)	3.93 (0.90)	−0.20 (0.61)	−0.26 (0.60)	−0.14 (0.63)
MIi	5.71 (0.72)	5.50 (0.84)	−0.27 (0.60)	−0.17 (0.38)	−0.16 (0.59)
GSE	3.86 (0.50)	3.87 (0.46)	−0.02 (0.26)	0.03 (0.24)	0.02 (0.29)
SSE	3.47 (0.47)	3.55 (0.43)	0.06 (0.26)	0.10 (0.26)	0.04 (0.25)
Control Traits	4.49 (0.97)	4.60 (0.91)	0.24 (0.51)	−0.06 (0.59)	0.14 (0.83)

*MSI, moral self-image; MIs, moral identity–symbolization; MIi, moral identity–internalization; GSE, generalized self-esteem; SSE, state self-esteem. For those variables with a significant omnibus ANOVA, means with different subscripts significantly differ at a p < 0.05*.

### Discussion

The goal of Study 5 was to determine if the prompts used by Cornelissen et al. (2013) that altered people's immoral behavior actually changed their MSI. We indeed found that the recall of one's (im)moral behavior changed one's MSI in the predicted directions. Also as predicted, this recall did not affect people's assessment on the control traits or their moral identities—including their symbolic moral identity, internalized moral identity (which is inconsistent with Study 4 but consistent with initial predictions), state self-esteem (which, again, is inconsistent with what previous research has found, see Barkan et al., 2012), and generalized self-esteem.

These findings appear to be consistent with the theory of moral compensation as symbolic self-completion (Zhong and Liljenquist, 2006; Jordan et al., 2011). That is, moral actions (or recalled moral actions) raise people's MSI, thus allowing them to relax their strivings on subsequent moral tasks. Similarly, immoral actions (or recalled immoral actions) lower people's MSI, thus leading them to put greater effort into acting morally on subsequent tasks (Sachdeva et al., 2009).

## General discussion

While we have witnessed people's moral inconsistencies both in real life and experimental research (e.g., Monin and Miller, 2001; Zhong and Liljenquist, 2006; Sachdeva et al., 2009; Jordan et al., 2011), until now, there was no validated measure to empirically examine the impact of these inconsistencies on people's MSI nor to examine the potential psychological processes driving these inconsistencies. As we propose in the current manuscript, these moral behaviors impact people's MSI in positive and negative ways. And as others have demonstrated (e.g., Cornelissen et al., 2013), these effects on the MSI subsequently affect related moral behaviors; in other words, MSI is a malleable construct that helps explain (im)moral behavior, like generosity and dishonesty.

This investigation accomplished two important objectives. First, it developed a scale to measure the MSI and, in order to investigate its convergent and discriminant validity, conceptually and empirically compared it to related constructs, such as moral identity (Aquino and Reed, 2002) and self-esteem (Rosenberg, 1965). Despite its theoretical relationship to both moral identity and self-esteem, we found that the MSI was empirically distinct from these constructs (Studies 1 and 2). It should be acknowledged that we did not find a relationship between the MSI and generalized self-esteem in either Study 4 or 5, which is curious since we found a relationship in the previous three studies in which generalized self-esteem was administered. A potential reason for this may be the samples used. The studies in which we found relationships between the MSI and generalized self-esteem employed non-student American adult samples, whereas those that did not, used Dutch student samples. It is possible that that given the secularism of Western Europe (Berger et al., 2008), Dutch students did not feel a connection between the state of their MSI and their general self-image. It could also be an age-related effect, such that in early adulthood, one's perceived moral state feels fairly isolated from his or her general self-image. In order to understand these effects further, more in-depth exploration of this issue is needed. Second, and relatedly, while the MSI was affected by three sources of influence (Kernis and Goldman, 2003)—social comparison, explicit feedback, and one's own behavior—this feedback did not affect these other constructs (with the exception of internalized moral identity in Study 4).

The current research also has implications outside the laboratory. Specifically, it suggests that specific events and feedback from the environment can affect people's MSI. This means that events in in the social world, such as reflecting on one's moral or immoral behavior during an interaction, can affect how an individual perceives his or her moral self. It also means that feedback about one's moral or immoral behavior, which routinely comes from experiences such as organizational, school, or family life, can affect the state of one's MSI.

### Limitations and future directions

There are limitations of the current investigation that warrant acknowledgment. First, in Study 4 we found an effect of feedback on internalized moral identity; feedback that one had almost reached one's ideal moral self increased one's reported importance of possessing moral traits (i.e., internalized moral identity), whereas feedback that one had met his or her ideal moral self led the individual to decrease such reported importance. This finding was unexpected given that prior research found internalized moral identity to be a stable trait (Jordan et al., 2011), and it is conceptualized as such (Aquino and Reed, 2002; Aquino et al., 2009). It is possible that the aspirational-level phenomenon (Zhang et al., 2007) may explain this result; however, more research is needed to investigate this and other possible explanations, as we did not find this effect in Study 5. Relatedly, in Studies 3 through 5, in which we either manipulated feedback about people's moral selves or allowed people to reflect on their own moral behavior, we always placed the MSI scale before the other scales, as observing changes to the MSI constituted the main goal of these studies. Therefore, we wished to minimize and distractions for participants between the presentation of the manipulations and people's ratings of their moral selves. We acknowledge that this methodology may have biased the results in favor of finding changes to people's MSI rather than to the scales that came later in the line-up (e.g., moral identity or state self-esteem).

Second, it is possible that the traits we used to capture the MSI were not traits that universally corresponded to people's conceptualization of the ideal moral person. For example, there is evidence that the connection between work and morality is specific to cultures with puritanical, Calvinist origins (Uhlmann et al., 2011). Thus, the trait, *hardworking*, might not elicit a prototype of the moral person equally across cultures. Therefore, it is possible that not all people collectively viewed these nine traits as equivalently referential to the moral self. While we used diverse samples to demonstrate our results, from American adults to international students, future research is needed to understand cultural differences on the conceptualization of moral prototypes.

Third, although the MSI is a state scale, we did not instruct people to rate how they were feeling “right now”—that is, at the current moment. Thus, it is possible, that some people rated themselves on these traits based on how they felt about their MSI, in general. However, results from Studies 3 through 5 did demonstrate variance between pre- and post-tests of individuals' MSI, suggesting that they were rating themselves based on perceptions at the current moment. However, it also suggests that leaving this phrasing out of the scale's preamble meant that our results served as a conservative test of our theory and that bigger pre- to post-test discrepancies may have been found had we emphasized the construct's state nature in our phrasing. We encourage future researchers using the MSI scale to experiment with the use of the “right now” statement and explore how it affects participants' responses.

An additional future direction would be to investigate the interaction between MSI and moral identity. There is suggestive evidence that MSI might interact with moral identity to affect people's engagement in moral behavior. For example, it might be that only when internalized moral identity is high (that is, when a person highly values possessing moral traits) does a low MSI prompt moral behavior in order to restore the moral self. As Aquino et al. (2009) wrote, “someone whose self-definition is organized around a set of moral traits should be motivated to behave in a moral manner to maintain this self-conception” (p. 124). They also hypothesized that people with a lower internalized moral identity would not be prompted to show such restorative behaviors. That said, there may be some empirical difficulties in testing this hypothesis due to ceiling effects, as the mean internalized moral identity is consistently found to be quite high (e.g., a 4.6 on a five-point scale, Aquino and Reed, 2002, and a 6.28 on a seven-point scale, Reed et al., 2007).

## Conclusion

Thinking about countless societal examples, a person can be both a pillar of the community and a thief, engaging in reflections that likely both boost and lower the way she thinks about her moral self. The current investigation demonstrates that the MSI is malleable and also presents a way to gauge this malleability with the goal of providing researchers with a more nuanced understanding of the intersection between the moral self and moral behavior.

## Funding

This research was supported by a grant (#451-13-031) awarded to the second author by The Netherlands Organization for Scientific Research (NWO).

### Conflict of interest statement

The authors declare that the research was conducted in the absence of any commercial or financial relationships that could be construed as a potential conflict of interest.

## Footnotes

^1^The literature has used other labels for what we refer to as the moral self-image, from moral self-concept (Mazar et al., 2008; Sachdeva et al., 2009) to moral self-worth (Sachdeva et al., 2009).

^2^Cornelissen and colleagues cited an earlier version of this manuscript, which had been presented at the Association for Consumer Research conference in St. Louis, MI in 2011: *Rules or Consequences? The Role of Ethical Mind-Sets in Moral Dynamics*.

^3^The procedures for all five studies in this manuscript received approval from an institutional review board prior to data collection. All procedures complied with the rules regarding conducting research with human subjects proposed by the American Psychological Association.

^4^We also ran CFAs across Samples 1a and 1b. Specifically, to examine the veracity of our proposed model related to other plausible models, we used the confirmatory factor analysis function of LISREL 8.80 maximum likelihood estimation method. Model fit was assessed by the Root Mean Square Error of Approximation (RMSEA), the Normed-fit Index (NFI), and the Comparative Fit Index (CFI). In addition, competing models were compared to our proposed model by means of chi-square differences. For Sample 1a, in which we compared our proposed model in which MSI, the symbolic and internalized sub-dimensions of moral identity, general self-esteem, moral disengagement, sympathy, negative reciprocity norms, and religiosity were analyzed as distinct factors with three other models in which symbolic moral identity and generalized self-esteem, and moral disengagement were loaded on to the same factor as MSI. The eight-factor model demonstrated better fit [χ^2^(1924)=5519.58; RMSEA = 0.059 (0.057, 0.060); NFI = 0.93; CFI = 0.96] than all three other models [7-factor: Δχ^2^(7) = 1721.13; RMSEA = 0.071 (0.069, 0.073); NFI = 0.92; CFI = 0.95; 6-factor: Δχ^2^(13) = 10,878.27; RMSEA = 0.12 (0.12, 0.12); NFI = 0.88; CFI = 0.91; 5-factor: Δχ^2^(18) = 10,847.16; RMSEA = 0.12 (0.12, 0.12); NFI = 0.87; CFI = 0.90]. Similarly, for Sample 1b we compared an 8-factor model, which examined the MSI, the symbolic and internalized sub-dimensions of moral identity, generalized self-esteem, moral disengagement, religiosity, and positive and negative affect as distinct factors with five other models in which the MSI was loaded on to factor along with symbolic moral identity, generalized self-esteem, and positive affect. The 8-factor model demonstrated better fit [χ^2^(1741) = 4588.07; RMSEA = 0.057 (0.055, 0.059); NFI = 0.95; CFI = 0.95] than all comparison models [7-factor with MSI and symbolic MI loaded together: Δχ^2^(7) = 1769.47; RMSEA = 0.073 (0.071, 0.075); NFI = 0.90; CFI = 0.93; 7-factor with MSI and generalized self-esteem loaded together:Δχ^2^(7) = 3774.63; RMSEA = 0.087 (0.085, 0.089); NFI = 0.89; CFI = 0.92; 7-factor model with MSI and positive affect loaded together:Δχ^2^(7) = 3417.13; RMSEA = 0.085 (0.083, 0.087); NFI = 0.92; CFI = 0.92; 6-factor model: Δχ^2^(13) = 5721.57; RMSEA = 0.99 (0.97, 0.10); NFI = 0.89; CFI = 0.90; 5-factor model: Δχ^2^(18) = 10778.19; RMSEA = 0.12 (0.12, 0.13); NFI = 0.86; CFI = 0.86].

^5^Although, the degrees of freedom were equivalent for both models, you cannot test the significance of a chi-square value with a degrees of freedom equal to 0. Thus, we set this to “1,” which is a conservative test of our hypothesis.

^6^Six participants were excluded due to behavior during the lab session (e.g., could not understand English or a fire alarm occurred in the middle of the session, forcing the lab to be evacuated), three because they did not believe that the MIP was a real test, and four because they took the post-test but did not take the pre-test.

^7^Due to a programming error in which the pre-test was measured on a seven-point scale and the post-test was measured on a nine-point scale, all MSI scores were standardized prior to analyses.

^8^Seven participants were excluded because they only took the post-test, one because he/she only took the pre-test, one who took the pre-test after the post-test, one who took the pre-test multiple times, one who's pre-test to post-test difference score was 8 standard deviations above the mean, and four people due to worrisome behavior in the lab (e.g., could not understand the consent form in English, were caught talking on their cell phones in the lab cubicle).

^9^They also had people recall times when they violated or acted consistently with a moral rule. These recalls did not lead to compensatory effects on immoral behavior and the relationship was not mediated by the MSI.

^10^Note that the scale in the Cornelissen et al. (2013) paper was not created by these authors themselves, but was the MSI scale presented in the current manuscript (cited from a conference presentation of this scale; see Footnote 2).

^11^Four participants were excluded because they did not take the pre-test, one because he/she did not take the post-test, six people who took the pre-test significantly less than 15 h before the post-test, and one person due to worrisome behavior in the lab (i.e., read the study debriefing before going in for the post-test).

## References

[B1] Adler A. (2006). Fundamentals of individual psychology, in Readings in the Theory of Individual Psychology, eds Slavik S. Carlson J. (New York, NY: Routledge/Taylor & Francis Group), 33–43.

[B2] Ahmed S. A. Jackson D. N. (1979). Psychographics for social policy decisions: welfare assistance. J. Consumer Res. 5, 229–239. 10.1086/208735

[B3] Allport G. W. (1955). Becoming; Basic Considerations for a Psychology of Personality. New Haven, CT: Yale University Press.

[B4] Aquino K. Freeman D. Reed I. I. Americus Lim, V. K. G. Felps W. (2009). Testing a social-cognitive model of moral behavior: the interactive influence of situations and moral identity centrality. J. Pers. Soc. Psychol. 97, 123–141. 10.1037/a0015406 19586244

[B5] Aquino K. Reed A. (2002). The self-importance of moral identity. J. Pers. Soc. Psychol. 83, 1423–1440. 10.1037/0022-3514.83.6.1423 12500822

[B6] Aronson E. (2003). The Social Animal. New York, NY: Macmillan.

[B7] Barkan R. Ayal S. Gino F. Ariely D. (2012). The pot calling the kettle black: distancing response to ethical dissonance. J. Exp. Psychol. Gen. 141, 757–773. 10.1037/a0027588 22409664

[B8] Bazerman M. H. Gino F. (2012). Behavioral ethics: toward a deeper understanding of moral judgment and dishonesty. Ann. Rev. Law Soc. Sci. 8, 85–104. 10.1146/annurev-lawsocsci-102811-173815

[B9] Berger P. L. Davie G. Fokas E. (2008). Religious America, Secular Europe?: A Theme and Variation. Hampshire, UK: Ashgate Publishing, Ltd.

[B10] Brown J. D. (1993). Self-esteem and self-evaluation: feeling is believing, in Psychological Perspectives on the Self, Vol. 4, ed Suls J. (Hillsdale, NJ: Erlbaum), 27–58.

[B11] Brown L. B. (1962). A study of religious belief. Br. J. Psychol. 53, 259–272. 10.1111/j.2044-8295.1962.tb00832.x 13873692

[B12] Colby A. Kohlberg L. Gibbs J. Lieberman M. Fischer K. Saltzstein H. D. (1983). A longitudinal study of moral judgment. Monogr. Soc. Res. Child Dev. 48, 1–124. 10.2307/1165935

[B13] Cornelissen G. Bashshur M. R. Rode J. Menestrel M. L. (2013). Rules or consequences? the role of ethical mind-sets in moral dynamics. Psychol. Sci. 24, 482–488. 10.1177/0956797612457376 23447556

[B14] Crocker J. Wolfe C. T. (2001). Contingencies of self-worth. Psychol. Rev. 108:593. 10.1037/0033-295X.108.3.593 11488379

[B15] Damon W. Hart D. (1992). Self-understanding and its role in social and moral development, in Developmental Psychology: An Advanced Textbook, 3rd Edn., eds Bornstein M. H. Lamb M. E. (Hillsdale, NJ: Lawrence Erlbaum Associates), 421–464.

[B16] Deci E. L. Ryan R. M. (1995). Human autonomy: the basis for true self-Esteem, in Efficacy, Agency, and Self-Esteem, ed Kernis M. H. (New York, NY: Plenum Press), 31–49.

[B17] DePaulo B. M. Kashy D. A. Kirkendol S. E. Wyer M. M. Epstein J. A. (1996). Lying in everyday life. J. Pers. Soc. Psychol. 70:979. 10.1037/0022-3514.70.5.979 8656340

[B18] Dunning D. (2007). Self-image motives and consumer behavior: how sacrosanct self-beliefs sway preferences in the market place. J. Consum. Psychol. 17, 237–249. 10.1016/S1057-7408(07)70033-5

[B19] Edwards J. R. (2002). Alternatives to difference scores: polynomial regression analysis and response surface methodology, in Advances in Measurement and Data Analysis, eds Drasgow F. Schmitt N. W. San Francisco, CA: Jossey-Bass), 350–400.

[B20] Eisenberg N. (2000). Emotion, regulation, and moral development. Annu. Rev. Psychol. 51, 665–697. 10.1146/annurev.psych.51.1.665 10751984

[B21] Eisenberg N. Cumberland A. Guthrie I. K. Murphy B. C. Shepard S. A. (2005). Age changes in prosocial responding and moral reasoning in adolescence and early adulthood. J. Res. Adolesc. 15, 235–260. 10.1111/j.1532-7795.2005.00095.x 20592955 PMC2893741

[B22] Eisenberger R. Lynch P. Aselage J. Rohdieck S. (2004). Who takes the most revenge? Individual differences in negative reciprocity norm endorsement. Pers. Soc. Psychol. Bull. 30, 787–799. 10.1177/0146167204264047 15155041

[B23] Fiske S. T. Cuddy A. J. Glick P. Xu J. (2002). A model of (often mixed) stereotype content: competence and warmth respectively follow from perceived status and competition. J. Pers. Soc. Psychol. 82, 878–902. 10.1037/0022-3514.82.6.878 12051578

[B24] Fiske S. T. Xu J. Cuddy A. C. Glick P. (1999). (Dis)respecting versus (Dis)liking: status and interdependence predict ambivalent stereotypes of competence and warmth. J. Soc. Issues 55, 473–489. 10.1111/0022-4537.00128

[B25] Gino F. Ayal S. Ariely D. (2009). Contagion and differentiation in unethical behavior the effect of one bad apple on the barrel. Psychol. Sci. 20, 393–398. 10.1111/j.1467-9280.2009.02306.x 19254236

[B26] Goldstone R. L. Chin C. (1993). Dishonesty in self-report of copes made: moral relativity and the copy machine. Basic Appl. Soc. Psych. 14, 19–32. 10.1207/s15324834basp1401_2

[B27] Gouldner A. (1960). The norm of reciprocity: a preliminary statement. Am. Soc. Rev. 25, 161–178. 10.2307/2092623

[B28] Greenwald A. G. Farnham S. D. (2000). Using the implicit association test to measure self-esteem and self-concept. J. Pers. Soc. Psychol. 79:1022. 10.1037/0022-3514.79.6.1022 11138752

[B29] Heatherton T. F. Polivy J. (1991). Development and validation of a scale for measuring state self-esteem. J. Pers. Soc. Psychol. 60, 895–910. 10.1037/0022-3514.60.6.895

[B30] Higgins E. T. (1987). Self-discrepancy: a theory relating self and affect. Psychol. Rev. 94, 319–340. 10.1037/0033-295X.94.3.319 3615707

[B31] Hoffman M. L. (1975). Sex differences in moral internalization and values. J. Pers. Soc. Psychol. 32:720. 10.1037/0022-3514.32.4.720 1185510

[B32] Jaffee S. Hyde J. S. (2000). Gender differences in moral orientation: a meta-analysis. Psychol. Bull. 126, 703–726. 10.1037/0033-2909.126.5.703 10989620

[B33] Jones S. C. (1973). Self-and interpersonal evaluations: esteem theories versus consistency theories. Psychol. Bull. 79, 185. 10.1037/h0033957 4570401

[B34] Jordan J. Mullen E. Murnighan J. K. (2011). Striving for the moral self: the effects of recalling past moral actions on future moral behavior. Personal. Soc. Psychol. Bull. 37, 701–713. 10.1177/0146167211400208 21402752

[B35] Kernis M. H. Cornell D. P. Sun C. R. Berry A. Harlow T. (1993). There's more to self-esteem than whether it is high or low: the importance of stability of self-esteem. J. Person. Soc. Psychol. 65:1190. 10.1037/0022-3514.65.6.1190 8295118

[B36] Kernis M. H. Goldman B. M. (2003). Stability and variability in self-concept and self-esteem, in Handbook of Self and Identity, eds Leary M. R. Tangney J. P. (New York, NY: Guilford Press), 106–127.

[B37] Kernis M. H. Johnson E. K. (1990). Current and typical self-appraisals: differential responsiveness to evaluative feedback and implications for emotions. J. Res. Pers. 24, 241–257. 10.1016/0092-6566(90)90019-3

[B38] Kohlberg L. (1971). Stages of moral development. Moral Educ. 23–92. 26578420

[B39] Kohlberg L. (1994). Stage and sequence: the cognitive-developmental approach to socialization, in Defining Perspectives in Moral Development, ed Puka B. (New York, NY: Garland Publishing), 1–134.

[B40] Kohlberg L. Levine C. Hewer A. (1983). Moral Stages: A Current Formulation and a Response to Critics. New York, NY; Karger.

[B41] Kotter J. P. (1973). The psychological contract: managing the joining-up process. Calif. Manage. Rev. 15, 91–99. 10.2307/41164442

[B42] Lapsley D. K. Lasky B. (2001). Prototypic moral character. Identity An Int. J. Theory Res. 1, 345–363. 10.1207/S1532706XID0104_03

[B43] Lewicki P. (1983). Self-image bias in person perception. J. Pers. Soc. Psychol. 45:384 10.1037/0022-3514.45.2.384 2778628

[B44] Mazar N. Amir O. Ariely D. (2008). The dishonesty of honest people: a theory of self-concept maintenance. J. Market. Res. 45, 633–644. 10.1509/jmkr.45.6.633

[B45] Mazar N. Zhong C. B. (2010). Do green products make us better people? Psychol. Sci. 21, 494–498. 10.1177/0956797610363538 20424089

[B46] McCarthy J. D. Hoge D. R. (1982). Analysis of age effects in longitudinal studies of adolescent self-esteem. Dev. Psychol. 18:372 10.1037/0012-1649.18.3.372

[B47] Monin B. Jordan A. H. (2009). The dynamic moral self: a social psychological perspective, in Personality, Identity, and Character: Explorations in Moral Psychology, eds Narvaez D. Lapsley D. K. (New York, NY: Cambridge University Press), 341–354.

[B48] Monin B. Miller D. T. (2001). Moral credentials and the expression of prejudice. J. Pers. Soc. Psychol. 81, 33–43. 10.1037/0022-3514.81.1.33 11474723

[B49] Moore C. (2008). Moral disengagement in processes of organizational corruption. J. Bus. Ethics 80, 129–139. 10.1007/s10551-007-9447-8

[B50] Moore C. Detert J. R. Klebe Treviño L. Baker V. L. Mayer D. M. (2012). Why employees do bad things: moral disengagement and unethical organizational behavior. Pers. Psychol. 65, 1–48. 10.1111/j.1744-6570.2011.01237.x

[B51] Moore C. Gino F. (2013). Ethically adrift: how others pull our moral compass from true north, and how we can fix it. Res. Organ. Behav. 33, 53–77. 10.1016/j.riob.2013.08.001

[B52] Moretti M. M. Higgins E. T. (1990). Relating self-discrepancy to self-esteem: the contribution of discrepancy beyond acutal-self ratings. J. Exp. Soc. Psychol. 26, 108–123. 10.1016/0022-1031(90)90071-S

[B53] Mulder L. B. Aquino K. (2013). The role of moral identity in the aftermath of dishonesty. Organ. Behav. Hum. Decis. Process. 121, 219–230. 10.1016/j.obhdp.2013.03.005

[B54] Murnighan J. K. Kim J. W. Metzger A. R. (1993). The volunteer dilemma. Adm. Sci. Q. 38, 515–538. 10.2307/2393335

[B55] Pelham B. W. (1995). Self-investment and self-esteem: evidence for a Jamesian model of self-worth. J. Pers. Soc. Psychol. 69:1141 10.1037/0022-3514.69.6.1141

[B56] Reed A. Aquino K. Levy E. (2007). Moral identity and judgments of charitable behaviors. J. Mark. 71, 178–193. 10.1509/jmkg.71.1.178

[B57] Rosenberg M. (1965). Society and the Adolescent Self-image. Princeton, NJ: Princeton University Press.

[B58] Rosenberg M. (1979). Conceiveing the Self. New York, NY: Basic Books.

[B59] Rosenberg M. (1986). Self-concept from middle childhood through adolescence, in Psychological Perspectives on the Self, Vol. 2, eds Suls J. Greenwald G. (Hillsdale, NJ: Erlbaum), 107–136.

[B60] Sachdeva S. Iliev R. Medin D. L. (2009). Sinning saints and saintly sinners: the paradox of moral self-regulation. Psychol. Sci. 20, 523–528. 10.1111/j.1467-9280.2009.02326.x 19320857

[B61] Schweitzer M. E. Ordonez L. Douma B. (2004). Goal setting as a motivator of unethical behavior. Acad. Manag. J. 47, 422–432. 10.2307/20159591

[B62] Shalvi S. Dana J. Handgraaf M. J. De Dreu C. K. (2011). Justified ethicality: observing desired counterfactuals modifies ethical perceptions and behavior. Organ. Behav. Hum. Decis. Process. 115, 181–190. 10.1016/j.obhdp.2011.02.001

[B63] Shalvi S. Gino F. Barkan R. Ayal S. (2015). Self-serving justifications doing wrong and feeling moral. Curr. Dir. Psychol. Sci. 24, 125–130. 10.1177/0963721414553264

[B64] Steele C. M. (1988). The psychology of self-affirmation: sustaining the integrity of the self, in Advances in Experimental Social Psychology, Vol. 21, Social Psychological Studies of the Self: Perspectives and Programs, ed Berkowitz L. (San Diego, CA: Academic Press), 261–302.

[B65] Uhlmann E. L. Poehlman T. A. Tannenbaum D. Bargh J. A. (2011). Implicit puritanism in american moral cognition. J. Exp. Soc. Psychol. 47, 312–320. 10.1016/j.jesp.2010.10.013

[B66] Wark G. R. Krebs D. L. (1996). Gender and dilemma differences in real-life moral judgment. Dev. Psychol. 32, 220–230. 10.1037/0012-1649.32.2.220

[B67] Watson D. Clark L. A. Tellegen A. (1988). Development and validation of brief measures of positive and negative affect: the PANAS scales. J. Pers. Soc. Psychol. 54, 1063–1070. 10.1037/0022-3514.54.6.1063 3397865

[B68] White R. D. (1999). Are women more ethical? recent findings on the effects of gender upon moral development. J. Public Admin. Res. Theory 9, 459–472. 10.1093/oxfordjournals.jpart.a024418

[B69] Wicklund R. A. Gollwitzer P. M. (1981). Symbolic self-completion, attempted influence, and self-deprecation. Basic Appl. Soc. Psych. 2, 89–114. 10.1207/s15324834basp0202_2

[B70] Wylie R. C. (1974). The Self-Concept: Theory and Research on Selected Topics, Vol. 2. Lincoln, NB: University of Nebraska Press.

[B71] Zhang Y. Fishbach A. Kruglanski A. W. (2007). The dilution model: how additional goals undermine the perceived instrumentality of a shared path. J. Pers. Soc. Psychol. 92:389. 10.1037/0022-3514.92.3.389 17352599

[B72] Zhong C.-B. Liljenquist K. (2006). Washing away your sins: threatened morality and physical cleansing. Science 313, 1451–1452. 10.1126/science.1130726 16960010

