# Supplementary material for: The Moral Self-Image Scale: Measuring and Understanding the Malleability of the Moral Self
Source: Front Psychol. 2015 Dec 15;6:1878. doi: 10.3389/fpsyg.2015.01878 (PMC4678225; doi:10.3389/fpsyg.2015.01878)
Supplement: Supplementary file 1 [file DataSheet1.docx]

**Appendix A: Moral Self-image Scale**

Please respond to the following statements as they apply to you.

1. Compared to the caring person I want to be, I am:

| 1 | 2 | 3 | 4 | 5 | 6 | 7 | 8 | 9 |
| --- | --- | --- | --- | --- | --- | --- | --- | --- |
| *Much less caring than the person I want to be* |  |  |  | *Exactly as caring as the person I want to be* |  |  |  | *Much more caring than the person I want to be* |

2. Compared to the compassionate person I want to be, I am:

| 1 | 2 | 3 | 4 | 5 | 6 | 7 | 8 | 9 |
| --- | --- | --- | --- | --- | --- | --- | --- | --- |
| *Much less compassionate than the person I want to be* |  |  |  | *Exactly as compassionate as the person I want to be* |  |  |  | *Much more compassionate than the person I want to be* |

3. Compared to the fair person I want to be, I am:

| 1 | 2 | 3 | 4 | 5 | 6 | 7 | 8 | 9 |
| --- | --- | --- | --- | --- | --- | --- | --- | --- |
| *Much less fair than the person I want to be* |  |  |  | *Exactly as fair as the person I want to be* |  |  |  | *Much more fair than the person I want to be* |

4. Compared to the friendly person I want to be, I am:

| 1 | 2 | 3 | 4 | 5 | 6 | 7 | 8 | 9 |
| --- | --- | --- | --- | --- | --- | --- | --- | --- |
| *Much less friendly than the person I want to be* |  |  |  | *Exactly as friendly as the person I want to be* |  |  |  | *More friendly than the person I want to be* |

5. Compared to the generous person I want to be, I am:

| 1 | 2 | 3 | 4 | 5 | 6 | 7 | 8 | 9 |
| --- | --- | --- | --- | --- | --- | --- | --- | --- |
| *Much less generous than the person I want to be* |  |  |  | *Exactly as generous as the person I want to be* |  |  |  | *Much more generous than the person I want to be* |

6. Compared to the hard-working person I want to be, I am:

| 1 | 2 | 3 | 4 | 5 | 6 | 7 | 8 | 9 |
| --- | --- | --- | --- | --- | --- | --- | --- | --- |
| *Much less hard-working than the person I want to be* |  |  |  | *Exactly as hard-working as the person I want to be* |  |  |  | *Much more hard-working than the person I want to be* |

7. Compared to the helpful person I want to be, I am:

| 1 | 2 | 3 | 4 | 5 | 6 | 7 | 8 | 9 |
| --- | --- | --- | --- | --- | --- | --- | --- | --- |
| *Much less helpful than the person I want to be* |  |  |  | *Exactly as helpful as the person I want to be* |  |  |  | *Much more helpful than the person I want to be* |

8. Compared to the honest person I want to be, I am:

| 1 | 2 | 3 | 4 | 5 | 6 | 7 | 8 | 9 |
| --- | --- | --- | --- | --- | --- | --- | --- | --- |
| *Much less honest than the person I want to be* |  |  |  | *Exactly as honest as the person I want to be* |  |  |  | *Much more honest than the person I want to be* |

9. Compared to the kind person I want to be, I am:

| 1 | 2 | 3 | 4 | 5 | 6 | 7 | 8 | 9 |
| --- | --- | --- | --- | --- | --- | --- | --- | --- |
| *Much less kind than the person I want to be* |  |  |  | *Exactly as kind as the person I want to be* |  |  |  | *Much more kind than the person I want to be* |
